# Accuracy of a “Single Question Nocturia Score” compared to the “International Prostate Symptoms Score” in the evaluation of lower urinary tract symptoms in benign prostatic hyperplasia: A study performed at Ndola Teaching Hospital, Ndola, Zambia

**DOI:** 10.1371/journal.pone.0198096

**Published:** 2018-06-26

**Authors:** Teddy Kajimotu, Kasonde Bowa

**Affiliations:** 1 Medical Student, Copperbelt University School of Medicine, Ndola, Zambia; 2 Professor of Urology, Copperbelt University School of Medicine, Ndola, Zambia; University Medical Center Utrecht, NETHERLANDS

## Abstract

**Introduction:**

The International Prostate Symptom Score (IPSS) is a useful tool approved by the World Health Organisation and the American Urological Association to measure the severity of lower urinary tract symptoms (LUTS). Although commonly used in urological practice, the IPSS has faced many challenges in terms of its usage in developing countries. In our setting, most patients presenting with this condition are elderly patients with a low literacy level. Given this background, the IPSS could be time consuming and difficult to administer to such patients and it may lead to additional costs to the services.

**Objectives:**

The objective of this study was to compare the accuracy of a Single Question Nocturia Score (SQNS) with the IPSS in evaluation of lower urinary tracts symptoms in men with Benign prostatic hyperplasia(BPH).

**Methods:**

The study was designed as a cross-sectional study using researcher-administered IPSS and SQNS questionnaires. The sensitivity, specificity, and correlation coefficient were used to compare the results obtained. Sensitivity was defined as the ability of the SQNS to detect severe-to-moderate symptoms, whereas specificity was defined as the ability of the SQNS to detect mild-to-no symptoms.

**Results:**

We recruited 162 patients with an age range between 50 and 88 years. The mean age was 66.7 (standard deviation ± 8.97 years). The IPSS showed that 85 patients (52%) presented with mild symptoms, and 77 patients (48%) presented with severe-to-moderate symptoms. In contrast, the SQNS showed that 88 patients (54.3%) presented with mild symptoms and 74 patients (46.7%) presented with severe-to-moderate symptoms. The sensitivity of the SQNS was 91%, and the specificity was 87%. The correlation coefficient of the SQNS to IPSS using Pearson correlation coefficient, was 0.74. This study showed that in our setting, the SQNS may be used as an alternative to the IPSS in assessing the severity of LUTS in men with BPH especially in a busy clinic.

## Introduction

The International Prostate Symptom Score (IPSS) was introduced in 1992[1,2]. The IPSS score has not been widely used in Africa owing to several factors including the availability of fewer specialists, short patient contact time, low literacy levels and visual problems in elderly patients, which prevents easy administration of the IPSS questionnaire[3,4].

The IPSS has been shown to be a highly sensitive and specific tool, with studies showing that these range from 55 to 75% [5,6]. Although widely used in routine Urology outpatient settings in developed countries, its use has been associated with several challenges, and the IPSS has required modifications to make it more sensitive, patient-friendly, and easier to administer. The IPSS has been translated into several languages to make it easier for non-English speaking patients to use[6,7]. Recent studies have shown that removal of 3 items from the IPSS score, which were then incorporated into a UWIN (Urgency, Weak Stream, Incomplete emptying and Nocturia) score produced good results, and this was more acceptable to patients[8].

The objective of this study was to compare the accuracy of a Single Question Nocturia Score(SQNS) against the gold standard IPSS for the assessment of LUTS.

## Methods

The study was performed at the Ndola Teaching Hospital Urology Outpatient Clinic. The definition of a patient was any patient aged ≥ 50 years with clinical evidence of BPH and evidence of LUTS attributable to BPH. Exclusion criteria were: patients with urethral stricture and neurogenic bladder (identified after careful history and physical examination), and those with complications of BPH.

The study tools used were the standard IPSS and a SQNS (in English), which were administered to patients by researchers. Words that the patient found difficult to understand were explained in the local language. The SQNS questionnaire comprised the ‘Nocturia’ question that was separated from the standard IPSS questionnaire and administered independently to all patients. The SQNS question was administered in the same manner as it was framed in the IPSS questionnaire. For the purpose of validation, the IPSS and SQNS were initially pre-tested in patients aged ≥ 50 years visiting the hospital for any other reason other than BPH.

Both, the IPSS and SQNS were administered to the patient at the same sitting by the same researcher. The IPSS was always administered prior to the administration of the SQNS. Our study was performed between October 2016 and March 2017(6months period).

The sample size was calculated at a confidence of 95%, with a prevalence of 85% for clinic attendance for BPH patients. With an expected total clinic attendance of 960 over the study period, the sample size was computed at 163.

The study was approved by the Ethics Review Board of our hospital. Prior to recruitment, we ensured that all patients included in the study had provided appropriate informed consent. A research questionnaire was administered to each patient to obtain all vital epidemiological data from the patient. Because these tools were administered by a researcher (provider), the literacy levels of the patients and time taken to complete the questionnaire were not recorded.

The total score obtained with each tool was recorded for each patient and the patients’ total scores were grouped into mild and moderate-to-severe. With regard to the IPSS, all scores ≤ 7were grouped as mild, whereas all scores ≥ 8 were grouped as moderate-to-severe^1^. For SQNS, the score was grouped as mild if the score was ≤ 2 whereas any score of ≥ 3 was grouped as moderate to severe. The maximum score for the SQNS was 5. The data were coded and then entered into the Statistical Package for Social Sciences version 20 (SPSS v20) software with double entry verification. Sensitivity was defined as the ability of the SQNS to show moderate and severe LUTS when compared to the IPSS. Specificity was defined as the ability of the SQNS to show mild LUTS when compared to the IPSS. These ratios were computed as percentages. The correlation coefficient was computed to numerically represent the extent and direction of the relationship between the SQNS and the IPSS in this data set. The Pearson coefficient was computed using SPSS version 20.

## Result

**Fig 1:**
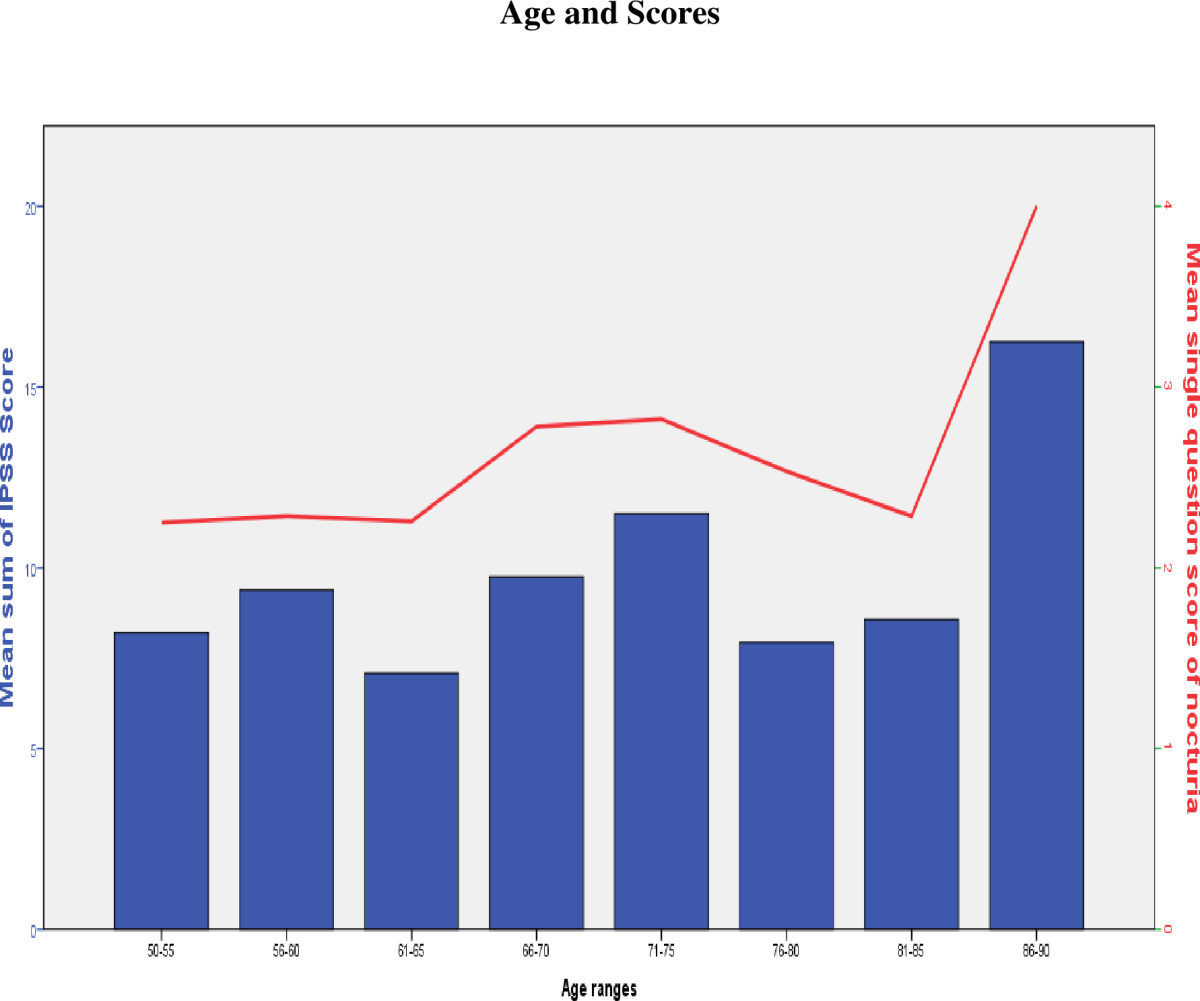
Shows the age group of the patient and the mean IPSS and SQNS per age group.

**Fig 2:**
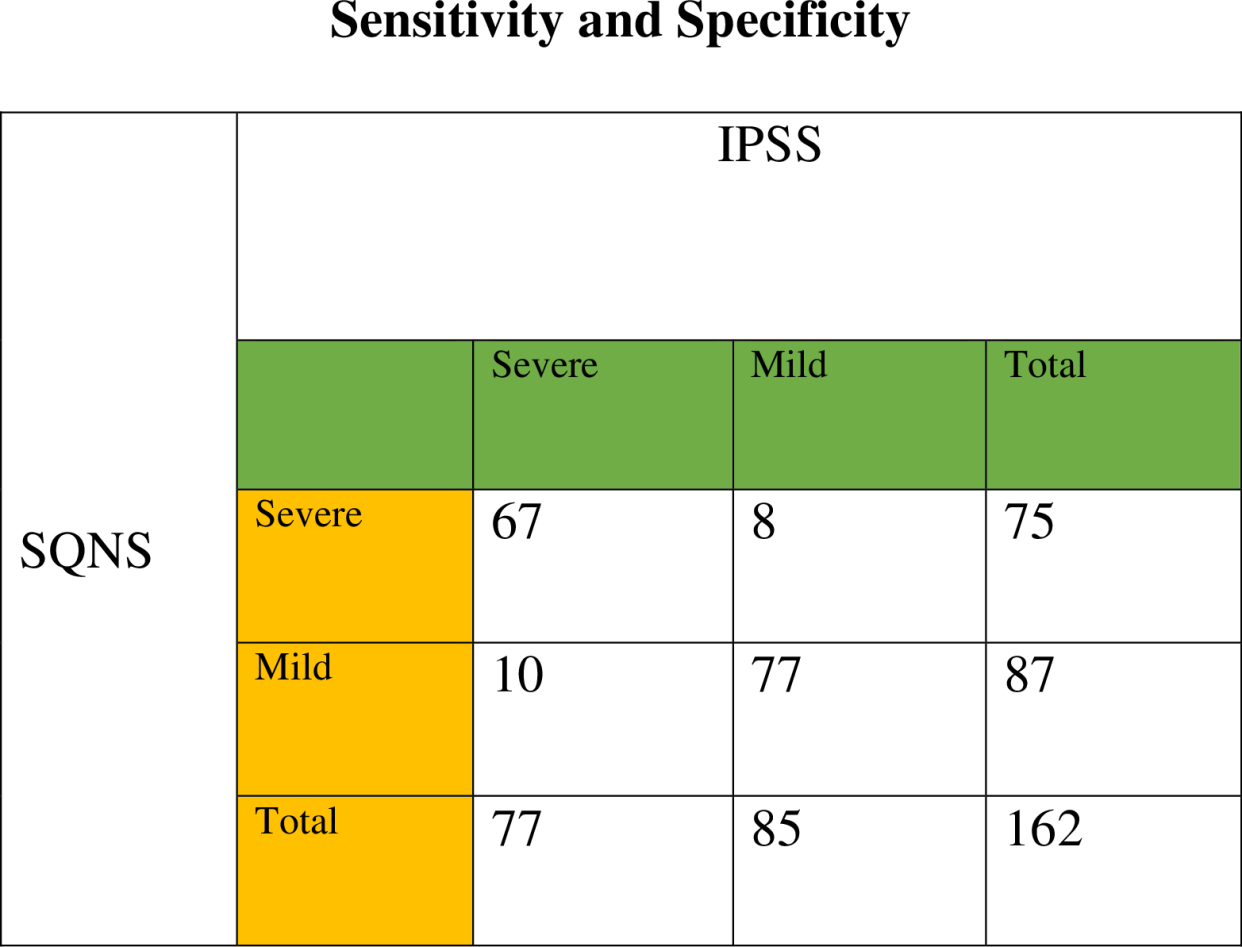
Shows the two by two table used to calculate the sensitivity and specificity of SQNS. Sensitivity=87%, Positive Predictive value=89%, Specificity=91%,Negative predictive vale=89%. Abbreviations: IPSS: International Prostate Symptom Score, SQNS: Single Question Noctura Score.

**Fig 3:**
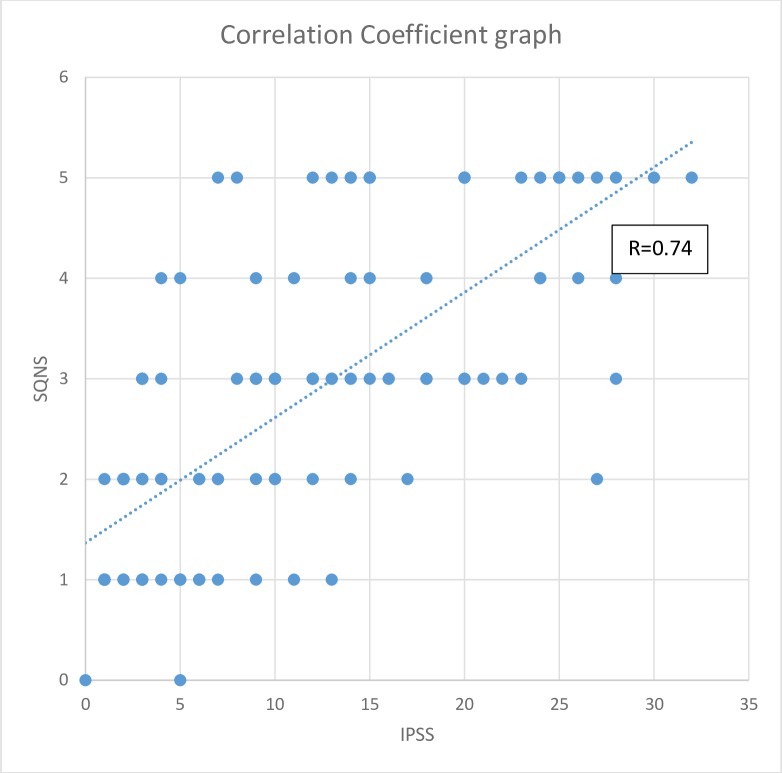
Shows the correlation coefficient of the SQNS relative to the IPSS. Pearson Correlation Co-efficient=0.74.

We recruited 165 patients; however, 3 were excluded due to non-availability of complete data—the age and a few components of the IPSS data were missing in these patients. Thus, 162 patients with a complete data set were included in the study. The age range was between 50 and 88 years with a mean age of 66.7 (standard deviation [SD] ± 8.97 years). This is shown in the bar graph in Fig 1. The results of Sensitivity and Specificity of the Single Question Nocturia Score is shown. in Fig 2. In Fig 3, a Scatterplot graph is used to show the correlation of SQNS and IPSS.

## Discussion

The IPSS results shown in this study, which evaluated the pattern of LUTS in BPH, is similar to other studies in Africa [3,4,5]. Ogwuche et al. studied the administration of IPPS in BPH. This study recruited 70 patients in Jos, Nigeria, and reported that the mean age of patients was 63 years at the time of presentation and most patients (59%) in their study showed moderate symptoms, whereas in our study most patients (53%) demonstrated mild symptoms. The low literacy levels in Africa have been shown to be a challenge for health care services in Africa[6]. In our study, this was offset by the researchers administering the questionnaire. The sustainability of this in a busy clinical setting would pose a major challenge[7,8].

Some researchers in developing countries have reported that the IPSS is difficult to use in developing countries, given the visual impairment and literacy levels in the target population [9,10,11]. However, Cams et al, have shown the usefulness of separating the individual questions and administering them independently. Also, Cams et al have shown that bothersome symptom when used alone, increase sensitivity of the tool by approximately 90% [12,13]. In a study assessing nocturia in patients diagnosed with BPH, Yoshimura et al. showed that nocturia is one of the most bothersome symptoms that patients easily recall. This symptom therefore lends itself to being used easily as a valid tool for the assessment of the severity of symptoms and the response to treatment[14].

To the best of the authors’ knowledge, this study was the first hospital based study to use a Single Question Nocturia Score(SQNS) in the assessment of LUTS. The findings of the current study show that the SQNS can be used in a low-resource setting to assess severity of men with LUTS due to BPH. There was a high sensitivity of 87% and a high specificity of 91% with the use of SQNS in this study; hence, SQNS can be used to assess severity of LUTS in patients with BPH. The high predictive value of 89% shows its accuracy in identifying the severity of symptoms which may be helpful in assessing LUTS from BPH in a busy Urology outpatient setting. The positive correlation coefficient of 0.74 suggests that it may be useful as an additional tool in outpatient clinics in Africa, or in other developing countries, with a limited human resources and facilities.

In conclusion, we recommend that the SQNS may serve as an accurate and convenient user-friendly tool in the management of BPH patients in high-volume low-resource settings.

Limitations: The questionnaires for the IPSS and SQNS in this study were administered at the same time. This may be a source of bias in the study.

## Supporting information

S1 Dataset IPSS data collection anonymized.xlsx. This is the study data in excel spread sheet without identifiers.
(XLSX)

S2 Dataset Clean data set IPSS vs SQs.sav.This is the study data in SPSS 20 format. (SAV)

## References

[1] Barry MJ, Fowler FJ Jr, O'Leary MP, Bruskewitz RC, Holtgrewe HL, Mebust WK et al The American Urological Association symptom index for benign prostatic hyperplasia. The Measurement Committee of the American Urological Association. The Journal of Urology. 1992;148(5):1549–57. 1279218 10.1016/s0022-5347(17)36966-5

[2] Mebust W, Roizo R, Schroeder F, Villers A. Correlations between pathology, clinical symptoms and the course of the disease. Proceedings of the international consultation on benign prostatic hyperplasia. 1991;26:53–62.

[3] Ogwuche EI, Dakum NK, Amu CO, Dung ED, Udeh E, Ramyil VM. Problems with administration of international prostate symptom score in a developing community. Annals of African Medicine. 2013;1:12(3):171 doi: 10.4103/1596-3519.117628 24005590 10.4103/1596-3519.117628

[4] Bowa K. International Prostate Symptoms Score usage in a developing country. Annals of African Medicine. 2013;1:12(3):174 24133758

[5] Dawam D, Rafindadi AH, Kalayi GD. Benign prostatic hyperplasia and prostate carcinoma in native Africans. BJU international. 2000 9 1;85(9):1074–7. 10848698 10.1046/j.1464-410x.2000.00677.x

[6] Omolewa M. Adult literacy in Africa: The push and pull factors. International Review of Education. 2008 11 1;54(5–6):697–711.

[7] O'Leary MP, Barry MJ, Fowler FJ Jr. Hard measures of subjective outcomes: validating symptom indexes in urology. The Journal of Urology. 1992;148(5):1546–8. 1279217 10.1016/s0022-5347(17)36965-3

[8] Badía X, García-Losa M, Dal-Ré R, Carballido J, Serra M. Validation of a harmonized Spanish version of the IPSS: evidence of equivalence with the original American scale. Urology. 1998;31:52(4):614–20. 9763080 10.1016/s0090-4295(98)00204-0

[9] Van der walt CL, Heyns CF, Edlin RS, Van Vuuren SP. Prospective comparison of a new visual prostate symptom score versus the international prostate symptom score in men with lower urinary tract symptoms. Urology. 2011;78:17–20. doi: 10.1016/j.urology.2011.01.065 21550646 10.1016/j.urology.2011.01.065

[10] Mallya A, Keshavamurthy R, Karthikeyan VS, Kumar S, Nagabhushana M, Kamath AJ. UWIN (Urgency, Weak stream, Incomplete Void, Nocturia) Score for Assessment of Lower Urinary Tract Symptoms: Could it Replace the American Urology Association Symptom Index Score? An Open Label Randomized Cross over Trial. LUTS: Lower Urinary Tract Symptoms. 2017 2 1. 10.1111/luts.12149 28256100

[11] Russo F, Di Pasquale B, Romano G, Vicentini C, Manieri C, Tubaro A, et al International prostate symptom score: Comparison of doctor and patient. Arch Ital Urol Androl. 1998;70 (3 Suppl):15–24. 9707766

[12] Boyle P. Cultural and linguistic validation of questionnaires for use in international studies: The nine-item BPH-specific quality-of-life scale. European Urology. 1997;32 (Suppl 2):50–2. 9248816

[13] Cam K, Senel F, Akman Y, Erol A. The efficacy of an abbreviated model of the International Prostate Symptom Score in evaluating benign prostatic hyperplasia. BJU international. 2003;1:91(3):186–9. 12581001 10.1046/j.1464-410x.2003.04055.x

[14] Yoshimura K, Ohara H, Ichioka K, Terada N, Matsui Y, Terai A et al Nocturia and benign prostatic hyperplasia. Urology. 2003;61(4):786–90. 12670566 10.1016/s0090-4295(02)02444-5

